# The role of CT chest in screening for asymptomatic COVID-19 infection in self-isolating patients prior to elective oncological surgery: findings from a UK Cancer Hub

**DOI:** 10.1259/bjr.20200994

**Published:** 2020-11-26

**Authors:** Derfel ap Dafydd, Michelle O’Mahony, Shaman Jhanji, Anand Devaraj, William Allum, David Nicol, Dominic M Blunt, Angela M Riddell

**Affiliations:** 1 Department of Radiology, The Royal Marsden NHS Foundation Trust, London, UK; 2 Department of Anaesthetics, The Royal Marsden NHS Foundation Trust, London, UK; 3 Department of Radiology, Royal Brompton and Harefield NHS Foundation Trust, London, UK; 4 Department of Surgery, The Royal Marsden NHS Foundation Trust, London, UK; 5 Department of Radiology, Imperial College Healthcare NHS, Hammersmith, London, UK

## Abstract

**Objectives:**

In accordance with initial guidance from the Royal College of Surgeons and Royal College of Radiologists, we evaluated the utility of CT of the chest in the exclusion of asymptomatic COVID-19 infection prior to elective cancer surgery on self-isolating patients during the pandemic.

**Methods:**

All surgical referrals without symptoms of COVID-19 infection in April and May 2020 were included. Patient records were retrospectively reviewed. Screening included CT chest for major thoracic and abdominal surgery. CTs were reported according to British Society of Thoracic Imaging guidelines and correlated with reverse transcriptase polymerase chain reaction (RT-PCR) and surgical outcomes.

**Results:**

The prevalence of RT-PCR confirmed COVID-19 infection in our screened population was 0.7% (5/681). 240 pre-operative CTs were performed. 3.8% (9/240) of CTs were reported as abnormal, only one of which was RT-PCR positive. 2% (5/240) of cases had surgery postponed based on CT results. All nine patients with CTs reported as abnormal have had surgery, all without complication.

**Conclusion:**

The prevalence of asymptomatic COVID-19 infection in our screened population was low. The pre-test probability of CT chest in asymptomatic, self-isolating patients is consequently low. CT can produce false positives in this setting, introducing unnecessary delay in surgery for a small proportion of cases.

**Advances in knowledge:**

Self-isolation, clinical assessment and RT-PCR are effective at minimising COVID-19 related surgical risk. The addition of CT chest is unhelpful. Our data have particular relevance during the second wave of infection and in the recovery phase.

## Introduction

The Royal Marsden Partners Cancer Hub became operational in late March 2020. As a ring-fenced “clean site”, its role was to enable cancer surgery for a regional network during the COVID-19 pandemic. As the height of the pandemic was imminent,^1^ concerns were raised regarding the risk of COVID-19-related post-surgical complications and mortality in elective surgical patients. Existing data included a retrospective series of surgical cases with confirmed COVID-19 infection, 280 of whom were elective cases; 53% (146/280) suffered post-operative pulmonary complications and 19% (53/280) died within 30 days of surgery.^2^ To add to these considerable surgical risks, underlying malignancy is also a recognised risk factor for severe infection and death from COVID-19.^3,4^

The CT features of COVID-19 infection and the role of CT chest in severe infection and emergency surgery are well established.^5–13^ However, there are limited data on the reliability of CT chest as a screening investigation for COVID-19 infection in asymptomatic individuals and in elective surgery. Data from the Diamond Princess cruise ship showed that positive CT findings may be present in as many as 54% (41/76) of asymptomatic contacts of confirmed carriers^12,14^ In a review of symptomatic patients with COVID-19 infection with initially false-negative reverse transcriptase polymerase chain reaction (RT-PCR), there were positive CT findings in 67% (10/15).^8^ Additionally, it has been reported that up to 50% of cases of COVID-19 infection are asymptomatic or infectious in the pre-symptomatic phase,^15,16^ while reported false-negative rates of RT-PCR are as high as 40%.^6,17^ Based on this collective literature, it is plausible that CT chest might identify a proportion of asymptomatic COVID-19 infections not detected by RT-PCR alone.

In view of these factors, The Royal College of Surgeons (RCS) and Royal College of Radiologists (RCR) published guidance on 15 April 2020 on the use of pre-operative CT chest together with RT-PCR in excluding COVID-19 infection prior to elective surgery during the pandemic.^18,19^ Its purpose was to minimise the risk of COVID-19-related complications and mortality following elective surgery (particularly major surgery, with anticipated high-dependency post-operative care) and to minimise the risk of transmission to other patients and staff.

Implementation of the guidance at The Cancer Hub gave us the opportunity to evaluate CT chest as part of pre-operative screening for COVID-19 infection, in order to clarify its role in minimising the associated surgical risk during the pandemic.

## Methods and materials

### Study design

This was a single centre, retrospective study conducted at a National Health Service (NHS) Cancer Hub during the COVID-19 pandemic. Medical and imaging records were reviewed of cases scheduled for surgery at the Cancer Hub in between 1 April 2020 and 31 May 2020. Institutional review board approval was obtained. Written, informed consent was waived.

### Participants

The study included all cases without symptoms of COVID-19 infection referred to the Cancer Hub for elective oncological surgery during the period of interest. Referrals included NHS England priority Level II (elective surgery with expectation of cure and required within 4 weeks) and priority Level III cases (elective surgery which can be delayed for 10–12 weeks).^20^

### Pre-operative screening

Screening was conducted either at The Royal Marsden Hospital (the principle site) or at The BUPA Cromwell Hospital (an independent sector site, affiliated for the duration of the Cancer Hub).

Prior to 21 April 2020, screening involved a telephone consultation, with the addition of RT-PCR in cases who were not self-isolating or who had a positive contact. From 21 April onwards, screening was carried out in accordance with RCS/RCR guidance and updated local Trust protocol.

The updated screening process included the following measures: a 7–14 day period of self-isolation; pre-operative clinical assessment to exclude symptoms or signs of COVID-19 infection; serological inflammatory marker assays; throat and nasopharyngeal RT-PCR within 2 days of surgery; CT chest within 2 days of surgery in high risk surgical cases.

According to Trust protocol, pre-operative CT chest was required for thoracic, upper gastrointestinal, lower gastrointestinal, hepatobiliary, pancreatic, and major head and neck surgery and any other cases likely to require Level II/III critical care post-operatively (*i.e.* HDU or ICU).

### Data collection

Results of investigations and clinical outcomes were obtained from Electronic Patient Records (EPR) and imaging archives. The following data items were extracted: basic demographics (age/ sex), surgical unit (*e.g.* thoracic), pre-operative RT-PCR result, pre-operative CT chest result, length of stay and any post-operative COVID-19 infections and related complications.

### Imaging technique

Unenhanced, high-resolution CT thorax was performed in the supine position. Mediastinal and lung algorithms were applied (Supplementary Material 1).

Supplementary Material 1.

Due to their self-isolating status, additional precautions were taken to minimise risk to the screened patients of contracting COVID-19 infection whilst in the radiology department. The screening CTs were scheduled for the beginning of the list on the “clean” scanner. Patients were provided with face masks and directed straight to the CT scanner, in order to avoid waiting areas. The scanner was cleaned before and after imaging.

### Imaging analysis

Screening CTs were reported prospectively within 4 hours of acquisition by appropriately trained radiologists. Imaging was interpreted according to British Society of Thoracic Imaging (BSTI) reporting guidance in COVID-19 infection (Table 1).^13^ Accordingly, findings were subclassified as “non-COVID”, “indeterminate for COVID-19”, “probable COVID-19” or “classic COVID-19”. For the purposes of the screening process and clinical risk stratification, cases reported prospectively as “non-COVID” were regarded as “negative” CT results. Cases reported prospectively as “indeterminate for COVID-19”, “probable COVID-19” and “classic COVID-19” were regarded as “positive” CT results. All cases reported prospectively as “indeterminate”, “probable COVID-19” or “classic COVID-19” were later reviewed retrospectively by a subspecialist in thoracic radiology of 14 years’ experience.

**Table 1.** British Society of Thoracic Imaging: Reporting guidance in suspected COVID-19 infection

Pattern	Description
CLASSIC COVID-19 (100% confidence for COVID)	Lower lobe predominant, peripheral predominant, multiple, bilateral foci of ground glass opacification +/− Crazy-paving Peripheral consolidation Air bronchograms Reverse halo/perilobular pattern
PROBABLE COVID-19 (71–99% confidence for COVID)	Lower lobe predominant mix of bronchocentric and peripheral consolidation Reverse halo/ perilobular pattern Ground glass opacities scarce
INDETERMINATE (<70% confidence for COVID)	Does not fit into definite, probable or Non-COVID Manifests above patterns, but the clinical context is wrong, or suggests an alternative diagnosis (*e.g.,* an interstitial lung disease in a connective tissue disease setting)
NON-COVID (70% confidence for alternative)	Lobar pneumonia Cavitating infections Tree-in-bud/centrilobular nodularity Lymphadenopathy, effusions Established pulmonary fibrosis

Subcategories of CT findings according to the BSTI guidance for Radiologists in COVID-19 infection^13^.

### Statistical analysis

The following data were obtained: Number/ percentage of pre-operative CT examinations deemed “abnormal” (by BSTI COVID-19 reporting guidance) and stratified by radiological subcategory (“indeterminate”, “probable COVID-19” and “classic COVID-19” patterns); concordance between CT findings and RT-PCR results; rates of delay or cancellation of surgery resulting from CT findings; and correlation of pre-operative CT findings with any post-operative COVID-19-related complications where applicable. For the purposes of statistical analysis, “positive” CTs subsequently classified on retrospective review by a thoracic radiologist as either “probable COVID-19” or “classic COVID-19” were regarded as true-positive CT results and the remainder regarded as false-positive CT results.

## Results

A total of 804 surgical referrals were processed through the Cancer Hub in between 1 April and 30 May 2020 (Table 2). Of these, 681 patients underwent pre-operative RT-PCR testing, 5 of whom (0.7%) had positive results – all 5 in the month of April 2020 (with no positive pre-operative RT-PCR tests in May 2020).

**Table 2.** Pre-operative screening activity by surgical unit

Surical unit	No of Patients	No of screening CTs done	No of patients operated on without CT screening	No of RT-PCR done	No of patients operated on without RT-PCR
Breast	219	2	217	173	46
Gynaecology	99	34	65	87	12
Head and Neck	42	7	35	41	1
LGI	119	77	42	99	20
Thoracics	82	67	15	77	5
HPB	38	28	10	38	0
Urology	93	12	81	79	14
Sarcoma	75	13	62	52	23
Endocrine	35	0	35	33	2
Plastics	2	0	2	2	0
Total	804	240 (30%)	564 (70%)	681 (85%)	123 (15%)

The table also includes patients who did not have RT-PCR testing, as a proportion of the referrals precede the introduction of the updated screening protocol on 21 April.

Over these 2 months, 240 (30%) of the Cancer Hub referrals underwent pre-operative CT chest (Table 3). There were 231 (96%) patients with “normal/ non-COVID” CT chest findings; one of whom was RT-PCR positive. The other 230 cases with “normal/ non-COVID” CT findings were RT-PCR negative.

**Table 3.** Summary of results of pre-operative screening chest CTs

CT result	Number
Normal/non-COVID	231 (96%)
Abnormal	9 (3.8%)
Probable/Classic COVID-19	3
Indeterminate	6
True +ve	3 (1.3%)
True –ve	230 (96%)
False + ve	6 (2.5%)
False –ve	1 (0.4%)

“Normal” and “abnormal CT” results are listed according to the original, prospective interpretation of the reporting radiologist. The CTs originally reported as “abnormal” were later retrospectively reviewed by a thoracic radiologist. Those CTs classified as “probable COVID” or “classic COVID” on retrospective review were considered to be True Positive CT results and likely attributable to COVID-19 infection (of unspecified age); the remainder were considered to be False Positive CT results.

**Figure 1.**
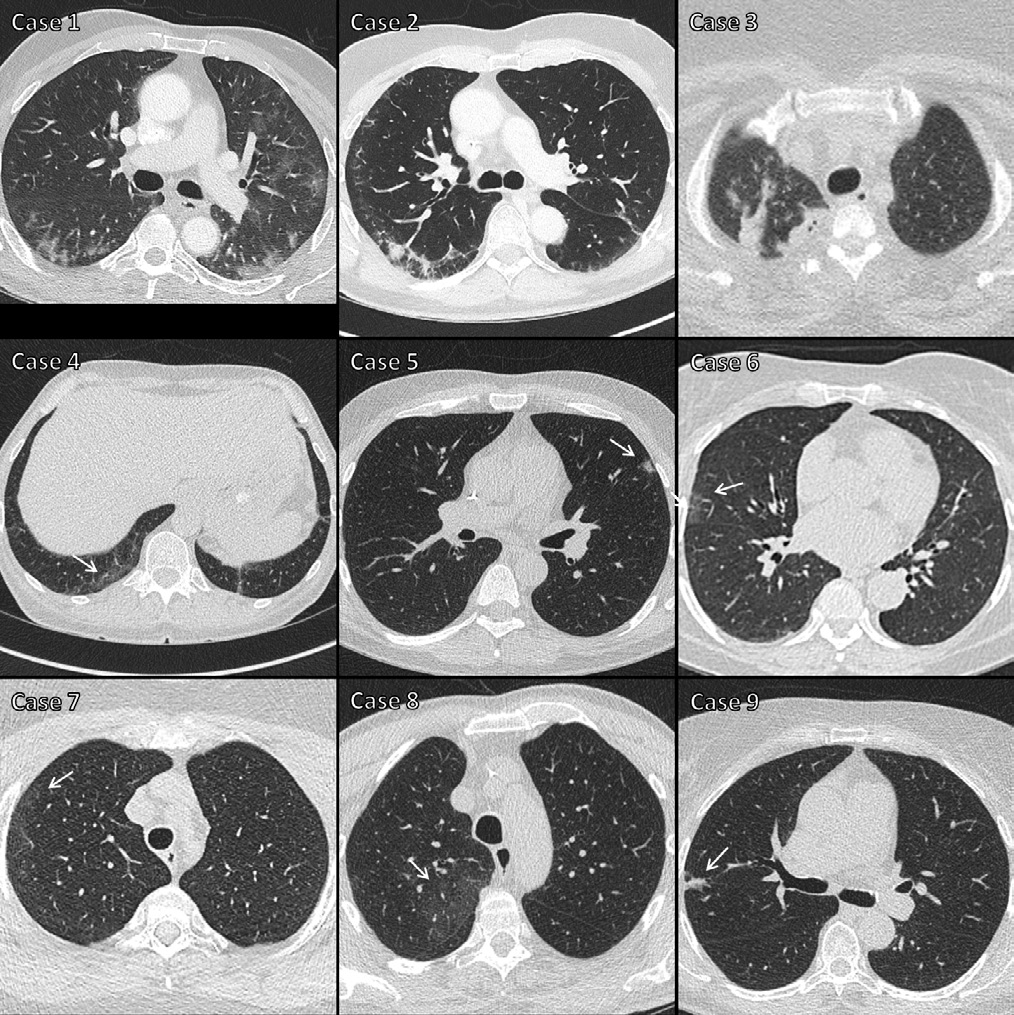
Images from the nine cases with “positive” CTs. The nine cases with “positive” CT results correspond to those listed in Table 4. Cases 1–3 were regarded as true-positive CT results, with areas of peripheral consolidation and/ or ground glass density, indicating either “probable COVID-19” or “classic COVID-19”. Cases 4–9 were regarded as false-positive CT results, with either very limited extent of ground glass opacity, or features more suggestive of a non-COVID-19 process (arrows).

**Figure 2.**
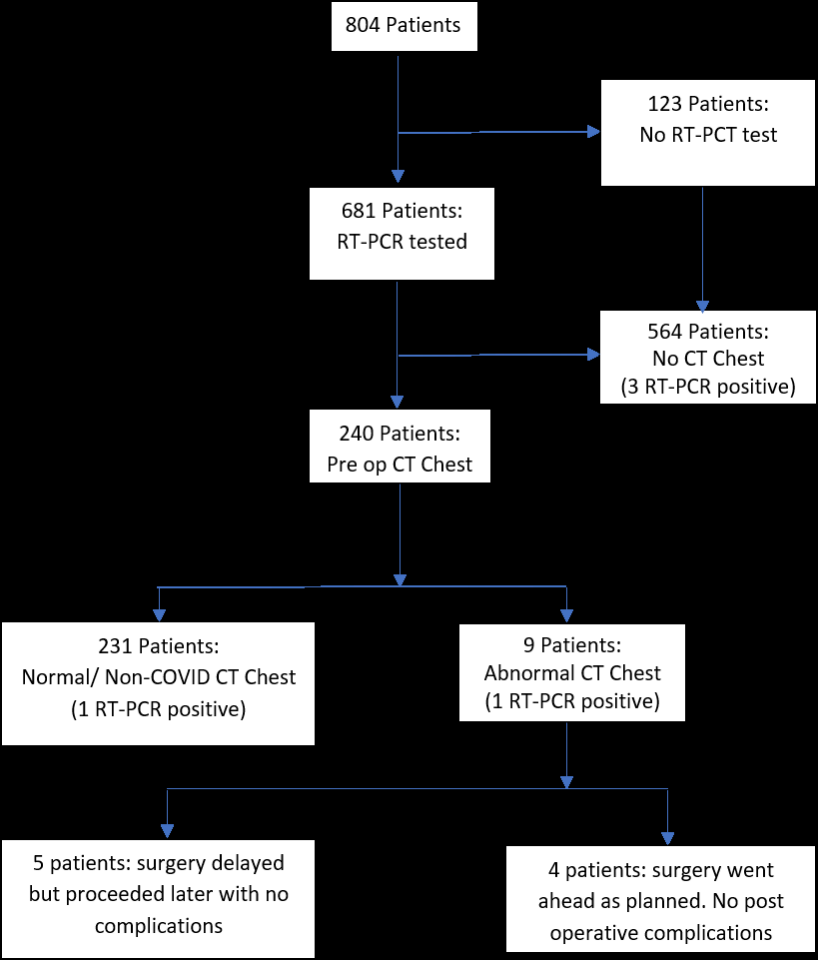
Flowchart showing the clinical impact of pre-operative CT chest on surgical outcome. RT-PCR, reverse transcriptase polymerase chain reaction

Nine patients (3.8%) had pre-operative CTs prospectively reported as abnormal (Table 4 and Figure 1). Five of these nine patients had their surgery postponed and had no post-surgical complications. Four others proceeded with surgery as originally planned and had no post-surgical complications (Figure 2).

**Table 4.** Planned surgery, COVID-19 screen results and surgical outcome in patients with abnormal pre-operative CT chest

#	Age/Sex	Planned surgery	RT-PCR	Pre-op CT – prospective report		Pre-op CT – retrospective review	Surgical outcome
1	64 M	Laparoscopic anterior resection	Positive	Probable COVID	TP	Probable COVID	Postponed 6 weeks. No complications.
2	66 M	Right hemicolectomy	Negative	Probable COVID	TP	Probable COVID	Postponed 6 weeks. No complications
3	55 F	Resection of thigh sarcoma	Negative	Indeterminate	TP	Probable COVID	Not postponed. No significant complications. Mild (non-COVID) post-op bacterial LRTI. LOS 8 days
4	62 F	Laparoscopic anterior resection	Negative	Probable COVID	FP	Non-COVID	Postponed 3 weeks. No complications
5	40 M	Defunctioning loop ileostomy	Negative	Indeterminate	FP	Indeterminate	Not postponed. No complications.
6	68 F	Hysterectomy, bilateral salpingoophorectomy and omentectomy	Negative	Indeterminate	FP	Indeterminate	Postponed 2 weeks. No complications.
7	69 F	Hysterectomy, bilateral salpingoophorectomy and omentectomy	Negative	Indeterminate	FP	Indeterminate	Not postponed. No complications.
8	76 M	Oesophagogastrectomy	Negative	Indeterminate	FP	Indeterminate	Not postponed. No complications.
9	79 F	Right hemicolectomy	Negative	Indeterminate	FP	Non-COVID	Postponed 1 month. No complications.

FP, false-positive CT result; LOS, length of stay; LRTI, lower respiratory tract infection; RT-PCR, reverse transcriptase-polymerase chain reaction; TP, true positive CT result.

TP and FP CT results were determined following retrospective review by a thoracic radiologist. Two cases categorised retrospectively as “probable COVID-19” were regarded as TPs based on high radiological confidence, but in view of static imaging findings and serially negative RT-PCR testing, they were interpreted as residual abnormalities from prior COVID-19 infection (rather than active infection).

Of the nine patients with prospectively reported abnormal CT findings, one was also RT-PCR positive. Although asymptomatic, this patient had features of “classic COVID-19” on CT. The patient’s surgery was postponed for 6 weeks, by which time the CT findings and RT-PCR had normalised and surgery proceeded without complication. The other eight patients with prospectively reported abnormal CTs were all RT-PCR negative, none of whom subsequently became RT-PCR positive or developed clinically suspected COVID-19 infection. There were two RT-PCR negative patients with CTs subclassified as “probable COVID-19” on retrospective review by a thoracic radiologist. In these two cases, given the lack of symptoms, negative serial RT-PCR testing, absence of more probable alternative CT diagnoses and static imaging findings, the CTs were interpreted as likely residual abnormalities from prior COVID-19 infection (rather than active COVID-19 infection). Both patients underwent surgery with no complications.

In April and May 2020, 70% (564/804) of the patients operated on at the Cancer Hub did not have a screening CT chest. None of them developed post-operative COVID-19 infection. One patient operated on at the Cancer Hub developed post-operative (RT-PCR confirmed) COVID-19 infection. The patient was transferred from another centre for palliative pleurodesis. The patient had a clear pre-operative CT chest and a negative pre-operative RT-PCR test.

## Discussion

Our study showed a low pre-test probability for pre-operative CT chest in asymptomatic COVID-19 infection. This was principally a reflection of the low number of COVID-19 infections in our screened population. At the height of the first wave of infection in the UK, the prevalence of RT-PCR confirmed COVID-19 infection in this asymptomatic, self-isolating cohort was very low at 0.7% (5/681). To put this into context, a previous large meta-analysis of 63 studies and 6218 patients suggests that at a COVID-19 prevalence of 1%, CT chest has a positive predictive value and negative predictive value of 1.5 and 99.8% respectively, representing a minimal diagnostic contribution.^21^

In our series 3.8% (9/240) of screening CTs were reported prospectively as abnormal. This is lower than the 7% rate of abnormal CTs reported in two other recent series (both in press at the time of writing).^22,23^ This may reflect differences in patient cohort in the study by Chetan et al, which included cases with and cases without symptoms of COVID-19 infection.^22^ There is also no indication as to whether the elective patients in that study self-isolated prior to surgery. In a recent RCR lead national audit of surgical patients who underwent screening CT chest during a 5-day period, follow-up data on clinical outcomes were unavailable in 25% (38/153) of cases.^23^ In contrast, our study includes at least a month’s follow-up in an exclusively asymptomatic, self-isolating cohort. Ours is the most complete data set of its kind and makes the strongest case so far against the routine use of screening CT chest in this context.

In our series, of the 240 patients who had a screening CT chest, there were only two true-positive cases who had their surgery postponed; one of which was RT-PCR positive and would have been postponed irrespective of the CT findings; the other was RT-PCR negative and most likely had CT stigmata of prior COVID-19 infection. Screening CT chest was not shown to have provided clear benefit in any case in our series.

The implications on surgical risk from residual CT abnormalities of past COVID-19 infection are not well understood. However, the two probable such cases in our series have since undergone surgery and neither had post-operative complications.

Although CTs subcategorised as “indeterminate” for COVID-19 infection are also of uncertain clinical significance, the four such cases (retrospectively subcategorised) in our series have also undergone surgery with no complications; only one of which was delayed. These may have represented cases of resolving or subclinical viral infection or alternative pneumonitides related to impaired immunity or neoadjuvant chemotherapy in the oncological setting. Careful clinical correlation and appropriate follow-up is important in assessing surgical risk in these indeterminate cases.

In our series, two CTs prospectively reported as abnormal were retrospectively recategorised as “non-COVID/ normal”. At the time of initial reporting, the radiologists were on high alert for any potentially subtle features of COVID-19 infection given the implicit risk to this pre-surgical cohort, and it is likely that this at least partly explains the inter-reader variability.

There is a potential risk to patients of contracting COVID-19 infection during radiological examination. The one case of post-operative COVID-19 infection in our series is a possible example of nosocomial transmission. Radiology departments experience high footfall of unwell patients and are front-facing services. It is recognised that live COVID-19 virus may persist on hard surfaces for up to 72 h.^24^ Radiology waiting areas and the CT scanner are therefore potentially high-risk vectors for transmission. This is an important consideration for patients who have otherwise been shielding to avoid infection prior to elective surgery.

We acknowledge certain limitations in this study. The retrospective design was unavoidable given the health-care emergency and urgent need for service reconfiguration and delivery. Within our cohort, there were only a small number of abnormal test events, precluding meaningful statistics on sensitivity and specificity. However, we feel the evidence provided from this study, together with the recently revised guidance from the RCS / RCR^18^ should give other centers sufficient confidence to proceed with elective surgery without the need for a pre-operative screening CT chest.

## Conclusion

To our knowledge, this is the first report of a formal screening protocol incorporating pre-operative CT chest in the detection of COVID-19 infection in an exclusively asymptomatic, self-isolating cohort prior to major oncological surgery. Given the very low prevalence (0.7%) of asymptomatic, RT-PCR confirmed COVID-19 infection in this series, the pre-test probability of CT chest was also low. Concordant with other emerging data,^22,25^ our study indicates that pre-operative self-isolation, clinical assessment and RT-PCR testing at a “clean site” are effective at minimising COVID-19 related surgical risk in elective cases. Pre-operative CT chest is unhelpful and may introduce unnecessary delay. These findings have particular relevance to this high clinical priority patient group during the current second wave of infection (and in the event of any subsequent waves) and during the recovery phase.
