# Supplementary material for: The role of CT chest in screening for asymptomatic COVID-19 infection in self-isolating patients prior to elective oncological surgery: findings from a UK Cancer Hub
Source: Br J Radiol. 2020 Nov 26;94(1117):20200994. doi: 10.1259/bjr.20200994 (PMC7774707; doi:10.1259/bjr.20200994)
Supplement: Supplementary Material 1. [file bjr.20200994.suppl-01.docx]

**Appendix A. Non-contrast, high resolution CT chest protocol**

| **Parameter** | **Value** |
| --- | --- |
| Voltage | 120 KVp |
| Current | Automatic tube current modulation |
| Pitch | 1.2 |
| Matrix size | 512 x 512 |
| Slice thickness | 1 mm |
| Slice interval | 0.7 mm |
